# GNSS Precise Relative Positioning Using A Priori Relative Position in a GNSS Harsh Environment

**DOI:** 10.3390/s21041355

**Published:** 2021-02-14

**Authors:** Euiho Kim

**Affiliations:** Department of Mechanical & System Design Engineering, Hongik University, 94, Wausan-ro, Mapo-Gu, Seoul 04066, Korea; euihokim@hongik.ac.kr

**Keywords:** GNSS, relative position, vehicle formation

## Abstract

To enable Global Navigation Satellite System (GNSS)-based precise relative positioning, real-time kinematic (RTK) systems have been widely used. However, an RTK system often suffers from a wrong integer ambiguity fix in the GNSS carrier phase measurements and may take a long initialization time over several minutes, particularly when the number of satellites in view is small. To facilitate a reliable GNSS carrier phase-based relative positioning with a small number of satellites in view, this paper introduces a novel GNSS carrier phase-based precise relative positioning method that uses a fixed baseline length as well as heading measurements in the beginning of the operation, which allows the fixing of integer ambiguities with rounding schemes in a short time. The integer rounding scheme developed in this paper is an iterative process that sequentially resolves integer ambiguities, and the sequential order of the integer ambiguity resolution is based on the required averaging epochs that vary for each satellite depending on the geometry between the baseline and the double difference line-of-sight vectors. The required averaging epochs with respect to various baseline lengths and heading measurement uncertainties were analyzed through simulations. Static and dynamic field tests with low cost GNSS receivers confirmed that the positioning accuracy of the proposed method was better than 10 cm and significantly outperformed a conventional RTK solution in a GNSS harsh environment.

## 1. Introduction

Precise relative positioning is an important capability for the applications that involve the cooperation of multiple vehicles, and it has typically been achieved by using carrier-based differential Global Navigation Satellite Systems (GNSS), such as Real Time Kinematic (RTK) systems [1,2,3]. An RTK system consists of reference and rover receivers with high-grade antennas and can provide centimeter-level positioning accuracy by using double difference code and carrier phase measurements. An RTK system must solve integer ambiguities in carrier phase measurements, for instance, by using Least-squares Ambiguity Decorrelation Adjustment (LAMBDA) algorithms [4,5,6] or a PREcise and Fast Method of Ambiguity Resolution (PREFMAR) method [7,8]. The LAMBDA method has been widely used in GNSS communities and it consists of three steps including a float solution generation, a decorrelation of double difference measurements, and an integer ambiguity search. The more recent PREFMAR method does not require a float baseline solution with a variance-covariance matrix but uses the correlation characteristics of double difference measurements on two or three frequencies to resolve integer ambiguities. Since the ambiguity values would change when the cycle slips of a carrier phase occur, an RTK system should detect and repair cycle slips if possible. Although some GNSS receivers report the occurrence of cycle slips, more can be made by using dual or triple frequency carrier phase measurements through cycle slip detections [9,10,11]. The confidence level determination of the resolved integer ambiguity is another challenging problem. There are a number of various statistical methods based on ratio tests, F-distribution, t-distribution, and Chi-square distribution [12,13,14].

While a dual-frequency receiver with a high-grade antenna is typically employed for a reliable RTK performance, a single-frequency multi-constellation RTK has been actively researched. The single-frequency multi-constellation RTK uses double difference code and carrier phase measurements and could provide equivalent positioning accuracy to the dual-frequency RTK with a short baseline of up to 10 km or so [15,16,17,18,19,20]. Verhagen et al. investigated the single-frequency RTK integer ambiguity resolution and positioning performance for different GNSS configurations [15]. Robert et al. investigated single-frequency Global Positioning System (GPS)/BeiDou Navigation Satellite System (BeiDou) RTK positioning performance with low-cost Ublox receivers compared to dual-frequency GPS with survey-grade receivers [16,17]. Jackson et al. evaluated the commercial low-cost single-frequency RTK and found that many of them suffered from performance degradation and wrong integer ambiguity fixes particularly when using a patch antenna [18]. To improve the single-frequency RTK, Lee et al. proposed an adaptive GPS/Inertial Navigation System integration that estimated a double difference noise covariance matrix on-the-fly [19]. Furthermore, Liu proposed a Kalman filter with the partial ambiguity fix method [20].

There are also several algorithms that take advantage of a known geometry of two GNSS receivers for precise positioning or attitude estimation. For a GNSS-based attitude determination, the fixed length of a baseline is typically used as a constraint in integer ambiguity search strategies [21,22,23,24,25,26]. The author of [6,21] introduced a detailed description of a modified LAMBDA method for a so-called GNSS Compass. Giorgi et al. developed search and shrink strategies for a baseline-constrained LAMBDA method [22]. Wang et al. used a fixed baseline to reduce the integer ambiguity search spaces that had been first expanded due to an inaccurate float solution [23].

Liu et al. introduced a single-epoch single-frequency algorithm based on partial least-squares ambiguity decorrelation adjustment that employed baseline constraints [24]. The authors of [25] performed a similar research with GPS/Galileo multi-constellation for a bridge dynamic monitoring problem. More recently, the authors of [26] introduced a precise positioning method based on an array of GNSS reference receivers. The fixed baseline lengths as well as the orientations between the array of the reference receivers helped to improve a float solution, then the integer ambiguity solution was obtained using LAMBDA.

However, the RTK based on LAMBDA and baseline-constrained LAMBDA may result in large positioning errors up to several meters due to wrong integer fixes and may take a long time to first fix in a harsh environment for GNSS. The main cause is the small number of satellites commonly in view between two receivers [23]. This paper presents a novel GNSS carrier phase-based precise relative positioning method using a known baseline information of two GNSS receivers that could outperform the conventional RTK approaches in a GNSS harsh environment.

In this paper, the baseline length was assumed to be accurately known up to a couple of centimeters, and the baseline heading direction was assumed to be measured from magnetometers. This baseline information was used to estimate an a priori relative position and its associated uncertainty. The a priori relative position uncertainty was derived by parameterizing the baseline length, baseline length measurement error, heading measurement error, and height measurement error.

In general, the a priori relative position and its uncertainty can be used to resolve integer ambiguities by using rounding, bootstrapping, and LAMBDA methods [27,28]. In this paper, a sequential rounding scheme is proposed for an integer ambiguity resolution with the a priori relative position. While the a priori relative position is valid in the beginning of operation, the proposed rounding scheme first computes a required averaging time for double difference carrier phase measurements of each satellite. The required averaging time may vary for each satellite depending on the relationship between the direction of a user-to-satellite line-of-sight vector and the direction of the a priori relative position error. After resolving the integer ambiguities of all double difference measurements, precise relative positioning can perform as a conventional RTK. Therefore, GNSS receivers should maintain the a priori relative position until all of the integer ambiguities are resolved. The paper will show that this initialization time is a function of baseline lengths and the uncertainty of the a priori relative position. The paper will also show that the initialization time can be significantly shortened from using the proposed sequential rounding scheme.

Section 2 reviews the GNSS double difference measurements and introduces an integer ambiguity fix approach utilizing the known geometry of two Unmanned Aerial Vehicles (UAV) placed on a levelled launchpad. Section 3 analyzes the impact of the errors in the a priori relative position and the geometry of the satellites in view on the integer ambiguity resolution with rounding schemes. Section 4 introduces a sequential rounding scheme approach that may accelerate the integer ambiguity fix process in the presence of the a priori baseline relative position errors. Section 5 presents the results of the static and dynamic field test of the proposed method and compares them with an open source RTK solution using LAMBDA and baseline-constrained LAMBDA methods.

## 2. Integer Ambiguity Resolution with Rounding Using a Known Relative Position

The carrier phase measurements of GNSS satellites can be modeled as shown in [29,30]:

(1) $$\Phi =r+t_{u}-t^{s}-I+T+N\lambda +\varepsilon ,$$

where Φ is carrier phase measurements, *r* is the true range between a user and a satellite, and *t_u_* and *t^s^* are the receiver and satellite clock errors, respectively. *I* is an ionospheric delay and *T* is a tropospheric delay. *N* is an integer ambiguity. λ is the wavelength of the carrier phase. ε includes the multipath, thermal noises, and modeling errors in the carrier phase measurements. When two GNSS receivers, master (*M*) and follower (*F*), are within several kilometers, the double difference formulation of the satellites *k* and *l* can be modeled as

(2) $$\nabla \Delta \Phi_{MF}^{kl}=\Delta \Phi_{MF}^{k}-\Delta \Phi_{MF}^{l}=-(1_{M}^{k}-1_{M}^{l})\cdot x_{MF}+\nabla \Delta N^{kl}\lambda +\nabla \Delta \varepsilon^{kl},$$

where $\Delta \Phi_{MF}^{k}$ and $\nabla \Delta \Phi_{MF}^{kl}$ are the single and double difference carrier phase measurements, respectively, between the receivers *M* and *F*. $1_{M}^{k}$ is the direction cosine vector from the receiver M to satellite k in East-North-Up (ENU) coordinates. l indicates a pivot satellite. $x_{MF}$ is the relative position vector from the receiver *M* to the receiver *F* in ENU coordinates. $\nabla \Delta N^{kl}$ is the double differenced integer ambiguity, and $\nabla \Delta \varepsilon^{kl}$ includes the double difference noise and multipath. In a conventional RTK system, $\nabla \Delta N^{kl}$ is resolved using various algorithms, such as LAMBDA [4,5,6]. The LAMBDA method does not place any constraints on $x_{MF}$ and uses no a priori information of $x_{MF}$ when searching for the integer ambiguty values. If the length of $x_{MF}$ is fixed in some cases like GNSS attitude determination problems, this constraint is used in baseline-constrained LAMBDA methods to more efficiently search for the integer ambiguity values [21,22,23,24,25,26]. Through those algorithms, centimeter levels of positioning accuracy can be obtained. However, as stated before, the integer ambiguity is still challenging in a GNSS harsh environment. Unlike the prior arts, the paper aims to find the integer ambiguity values with an a priori relative position, which would provide a more accurate estimation of the integer ambiguity in a GNSS harsh environment.

Let us denote the true and estimated initial baseline as $x_{MF}$ and ${\hat{x}}_{MF}$, respectively. Then, ${\hat{x}}_{MF}$ can be modeled as

(3) $${\hat{x}}_{MF}=x_{MF}+b_{0},$$

where $b_{0}$ is the position bias of the known initial baseline. Using Equation (3), Equation (2) can be expressed as

(4) $$\nabla \Delta \Phi_{MF}^{kl}+(1_{M}^{k}-1_{M}^{l})\cdot {\hat{x}}_{MF}=(1_{M}^{k}-1_{M}^{l})\cdot b_{0}+\nabla \Delta N^{kl}\lambda +\nabla \Delta \varepsilon^{kl}.$$

**Figure 1:**
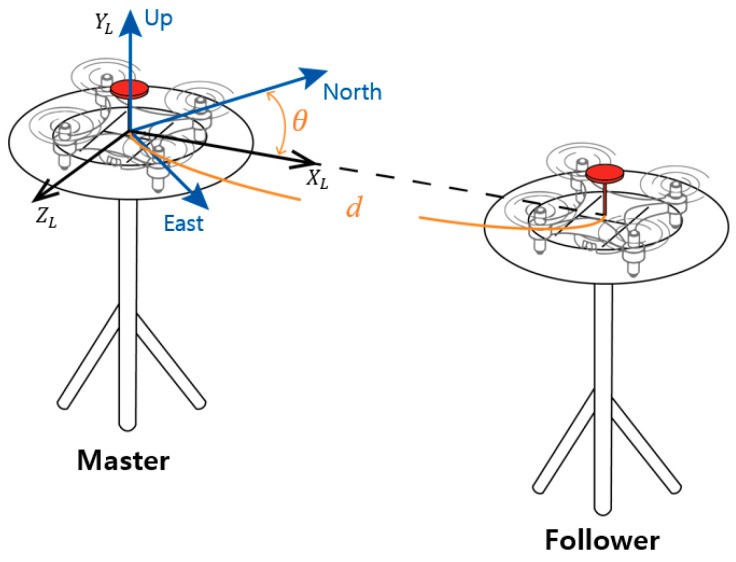
The proposed launchpad geometry. Two unmanned aerial vehicles (UAVs) are placed on the leveled pad discs and their heading directions are aligned with the baseline. The Master UAV is located at the origin of the local East-North-Up (ENU) coordinate system, while the Follower UAV is placed at [dsinθ,dcosθ,0].

In Equation (4), the double differenced integer ambiguities can be reliably and instantly measured if $b_{0}$ is sufficiently small. In practice, accurately surveyed locations will not be available at all times. A practical approach to obtain ${\hat{x}}_{MF}$ is to use a tool that allows a user to conveniently place two receivers at a known baseline length and height. As an example, Figure 1 shows two UAVs equipped with a GNSS receiver and a magnetometer on a launchpad. The GNSS receiver antenna is to be placed in the marked point on the launchpad, and the distance, d, between the marks is fixed. The origin of a local coordinate system, [X_L_, Y_L_, Z_L_], in the launchpad is located at the position of the master receiver. θ is the baseline heading or X_L_ direction with respect to North and is measured from the onboard magnetometers aligned with the baseline.

Assuming that the two GNSS receivers are in the same height above the ground and that there are no measurement errors inand d and θ, then the a priori relative position in ENU coordinates, ${\hat{x}}_{MF,aprioi}$, in a vector form is computed as

(5) $${\hat{x}}_{MF,aprioi}=[d\sin(\theta ),d\cos(\theta ),0].$$

Then, ${\hat{x}}_{MF,aprioi}$ in Equstion (5) can be substituted into Equation (2) and integer ambiguities can be found from a roundoff, such that

(6) $$\nabla \Delta {\hat{N}}^{kl}={\left[\frac{\nabla \Delta \Phi_{MF}^{kl}+(1_{M}^{k}-1_{M}^{l})\cdot {\hat{x}}_{MF,aprioi}}{\lambda }\right]}_{roundoff}={\left[\nabla \Delta N^{kl}+\frac{(1_{M}^{k}-1_{M}^{l})\cdot b_{0}+\nabla \Delta \varepsilon^{kl}}{\lambda }\right]}_{roundoff},$$

where ${\left[\;\right]}_{roundoff}$ is a roundoff operator.

The correctness of the $\nabla \Delta {\hat{N}}^{kl}$, of course, depends on the errors in the measurements of d and θ, which will be further discussed in the next section. After finding $\nabla \Delta {\hat{N}}^{kl}$ for *n*-1 satellites, the a-posteriori relative position computed from using GNSS measurements, ${\hat{x}}_{MF,GNSS}$, can be obtained from solving the following equation.

(7) $$\begin{array}{l} \underset{Y}{\underset{︸}{\left[\begin{array}{c} \nabla \Delta \Phi_{\mathrm{MF}}^{1l}-\nabla \Delta {\hat{N}}^{1l}\lambda \\ \nabla \Delta \Phi_{\mathrm{MF}}^{2l}-\nabla \Delta {\hat{N}}^{2l}\lambda \\ \vdots \\ \nabla \Delta \Phi_{\mathrm{MF}}^{nl}-\nabla \Delta {\hat{N}}^{nl}\lambda \end{array}\right]}}=\underset{G}{\underset{︸}{\left[\begin{array}{c} -(1_{M}^{1}-1_{M}^{l})\\ -(1_{M}^{2}-1_{M}^{l})\\ \vdots \\ -(1_{M}^{n}-1_{M}^{l}) \end{array}\right]}}\cdot x_{MF,GNSS}+\left[\begin{array}{c} \nabla \Delta \varepsilon^{1l}\\ \nabla \Delta \varepsilon^{2l}\\ \vdots \\ \nabla \Delta \varepsilon^{nl} \end{array}\right]\\ \;\;\;\;\;\;\;\;\;\;\mathrm{and}\\ \;\;\;\;\;{\hat{x}}_{MF,GNSS}={\left(G^{T}W^{-1}G\right)}^{-1}G^{T}W^{-1}Y \end{array}$$

where **W** is a weighting matrix. The superscript T indicates a transpose.

In the next section, the expression of ${\hat{x}}_{MF,aprioi}$ is derived with small measurement errors. Moreover, a metric that quantitatively evaluates the impact of the measurement errors on the integer ambiguity fix will be discussed.

## 3. Analysis on the Impact of Errors in the A Priori Relative Position

An integer ambiguity resolution would be a trivial problem if the relative position of the baseline between the two antennas was accurately measured. The impact of the measurement errors of *d,* θ, and height difference of the pads, *h*, on the integer ambiguity is analyzed as follows.

Let us introduce the additive measurement errors δd and δθ of the baseline length and heading, respectively. The height measurement error between the two pads is denoted as δh. Then, the estimated relative position in ENU coordinates can be expressed as

(8) $${\hat{x}}_{MF,aprioi}=[\hat{d}\sin(\hat{\theta })\cos(\hat{\alpha }),\hat{d}\cos(\hat{\theta })\cos(\hat{\alpha }),\hat{h}],$$

where $\hat{d}=d+\delta d$, $\hat{\theta }=\theta +\delta \theta$, $\hat{h}=h+\delta h$, and $\hat{\alpha }=\arcsin(\hat{h}/\hat{d})$. In practice, the baseline length error, δd and δh can be controlled better than one centimeter. The azimuth angle measurement errors of low-cost magnetometers have been reported as 0.25–2.0 degrees [31,32].

Assuming that δd,δθ, and,δh are small and δh is insignificant compared to $\hat{d}$, such that $\hat{\alpha }$ is also small, then

(9) $$\begin{array}{cc} \hat{d}\sin(\hat{\theta })\cos(\hat{\alpha }) & =(d+\delta d)(\sin(\theta )\cos(\delta \theta )+\cos(\theta )\sin(\delta \theta ))\cos(\hat{\alpha })\\ & \approx (d+\delta d)(\sin(\theta )+\delta \theta \cos(\theta ))\\ & \approx d\sin(\theta )+d\delta \theta \cos(\theta )+\delta d\sin(\theta ) \end{array}$$

and

(10) $$\begin{array}{cc} \hat{d}\cos(\hat{\theta })\cos(\hat{\alpha }) & =(d+\delta d)(\cos(\theta )\cos(\delta \theta )-\sin(\theta )\sin(\delta \theta ))\cos(\hat{\alpha })\\ & \approx (d+\delta d)(\cos(\theta )-\delta \theta \sin(\theta ))\\ & \approx d\cos(\theta )-d\delta \theta \sin(\theta )+\delta d\cos(\theta ). \end{array}$$

Thus, $b_{0}$ can be approaximated as

(11) $$b_{0}\approx \left[\begin{array}{c} d\delta \theta \cos(\theta )+\delta d\sin(\theta )\\ -d\delta \theta \sin(\theta )+\delta d\cos(\theta )\\ \delta h \end{array}\right].$$

Equation (11) indicates that the impact of the errors in ${\hat{x}}_{MF,aprioi}$ becomes small as the baseline length, *d*, decreases. Therefore, a shorter baseline length is preferred if the measurement errors cannot be maintained as small.

**Figure 2:**
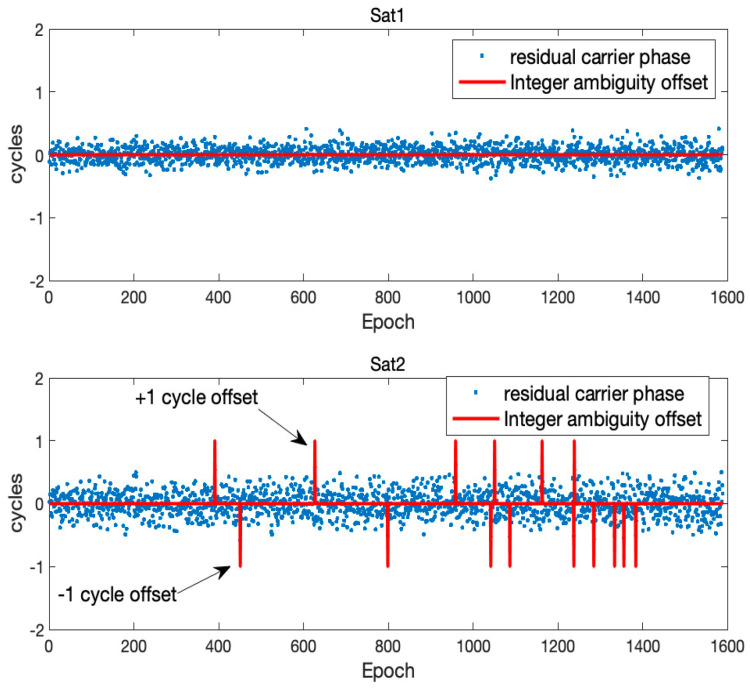
Two examples of residual double difference carrier phase measurements in cycles subtracted from the correct integer ambiguities. Satellite 1 (Sat1) has no wrong integer ambiguity offsets, but satellite 2 (Sat2) suffers from many wrong ones.

Figure 2 shows two examples, satellite 1 (Sat1) and satellite 2 (Sat2), of residual double difference carrier phase measurements in cycles subtracted from the correct integer ambiguities. The examples were generated using the SatNav simulator [33] with *d* as 2 m, and δd and δh were assumed to follow a zero-mean Gaussian distribution with standard deviations of 1 cm. δθ was assumed to follow a zero-mean Gaussian distribution with 1.0 degrees standard deviations. In Figure 2, the red line indicates the integer ambiguity offsets resulted from using Equation (6) with measurements at one epoch only. The standard deviations of the carrier phase residuals of Sat1 and Sat2 are 0.12 cycles and 0.19 cycles, and there are many cases of one cycle integer ambiguity offsets in Sat2.

**Figure 3:**
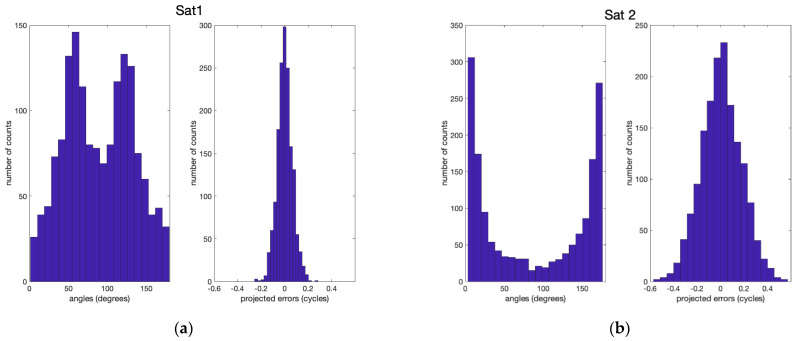
Statistics of the relative angles between the double difference line-of-sight vectors and $b_{0}$ are shown in the left column in (**a**) and (**b**) for satellite 1 and satellite 2, respectively. Their inner product values are shown in the right columns.

The reason for the relatively large residual carrier phase and many wrong integer ambiguities in Sat2 is that the direction of the double difference line-of-sight vector is almost parallel to the direction of $b_{0}$, which amplifies the overall error term in Equation (4). Figure 3 shows the statistics of the relative angles between the double difference line-of-sight vectors of Sat1 and Sat2 and $b_{0}$ in the left column. Figure 3 also shows the corresponding projected baseline errors onto the double difference line-of-sight vectors in cycles in the right column. Unlike Sat1, there is a significant number of relative angles close to 0 and 180 degrees in Sat2, which results in the much larger projected errors that could induce wrong integer fixes.

From the above analysis, it is possible to discern which satellite combinations would suffer from a wrong integer ambiguity fix by comparing the double difference line-of-sight vectors and $b_{0}$. However, $b_{0}$ is unknown and is a function of random variables of δd, δθ, and δh. As $b_{0}$ cannot be explicitly obtained, the impact of the measurement errors is predicted by using the baseline geometry and sensor measurement uncertainty in this paper.

Let us treat δd, δθ, and δh. as random variables with Gaussian distributions, such that $\delta d\sim N(0,\sigma_{\delta d})$, $\delta \theta \sim N(0,\sigma_{\delta \theta })$, and $\delta h\sim N(0,\sigma_{\delta h})$. With that, the variance of the vector of double difference carrier phase residuals in cycles, $\nabla \Delta \bar{N}$, after applying ${\hat{x}}_{MF,aprioi}$ can be modeled as from Equations (6) and (11).

(12) $$Var(\nabla \Delta \bar{N})=\frac{G\cdot P\cdot G^{T}+Var(\nabla \Delta \bar{\varepsilon })}{\lambda^{2}}$$

and

(13) $$P=Var\left(\left[\begin{array}{c} d^{2}\cos^{2}(\theta )\sigma_{\delta \theta }^{2}+\sin^{2}(\theta )\sigma_{\delta d}^{2}\\ d^{2}\sin^{2}(\theta )\sigma_{\delta \theta }^{2}+\cos^{2}(\theta )\sigma_{\delta d}^{2}\\ \sigma_{\delta h}^{2} \end{array}\right]\right),$$

where P is the covariance matrix of $b_{0}$ based on the baseline geometry and sensor measurement accuracies. Var( ) denotes the variance of the variable or matrix inside the parenthesis. $\nabla \Delta \bar{\varepsilon }$ is the vector of the double difference noise and multipath. The diagonal element of the $Var(\nabla \Delta \bar{N})$ can be used as a metric to discern troublesome satellites for a reliable integer ambiguity estimation. This can also be used to determine the number of averaging lengths to reduce the effect of $b_{0}$ in the next section.

## 4. Sequential Integer Ambiguity Resolution with Rounding

To reduce the impact of sensor measurement errors and noise, it is desirable to take the averages of multi-epoch measurements to compute integer ambiguities. This section first discusses how to determine an averaging time and proposes iterative methods that would help to reliably fix the integer ambiguities based on roundoff approaches.

From Equation (6), the averaged integer ambiguity estimate can be obtained from

(14) $$\nabla \Delta {\hat{N}}_{avg}^{kl}={\left[\frac{1}{\lambda N_{avg}^{kl}}\sum_{t=1}^{N_{avg}^{kl}}\nabla \Delta \Phi_{MF}^{kl}(t)-(1_{M}^{k}(t)-1_{M}^{l}(t))\cdot {\hat{x}}_{MF,apriori}(t)\right]}_{roundoff},$$

where $N_{avg}^{kl}$ is the number of an averaging epoch for the double difference measurements of *k* and *l* satellites. *t* indicates an epoch. $N_{avg}^{kl}$ is computed based on the uncertainty of a sample mean as follows:

(15) $$N_{avg}^{kl}={\left[\frac{Var{\left(\nabla \Delta \bar{N}\right)}_{\left(k,k\right)}}{\sigma_{\nabla \Delta {\hat{N}}_{avg}^{}}^{2}}\right]}_{round\text{-}up}.$$

$\sigma_{\nabla \Delta {\hat{N}}_{avg}^{}}$ is a desired standard deviation in estimating the double difference integer ambiguity and is conservatively set to 0.1 cycles in this paper. The subscript (*k*,*k*) denotes the *k*th row and *k*th column element. ${\left[\;\right]}_{round\text{-}up}$ is a round-up operator.

**Figure 4:**
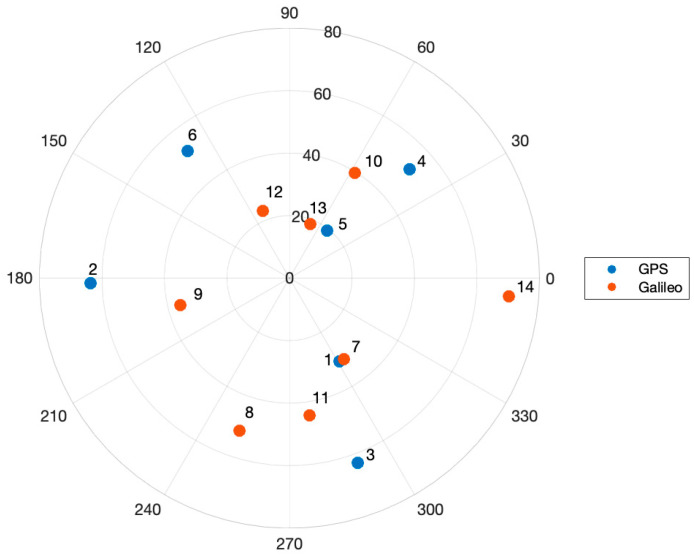
A sky plot of the simulated Global Positioning System (GPS)/Galileo constellation with respect to the user’s location. The dots represent a satellite and the numbers beside the dots indicate satellite numbers. The satellite constellation was generated using the SatNav simulator.

Based on Equations (12) and (13), $Var(\nabla \Delta \bar{N})$ would increase as the baseline length increases. To see the effects of the baseline length on the number of averaging epochs, a GPS/Galileo satellite constellation was simulated as shown in Figure 4. The baseline vector is toward the North, and five cases of baseline lengths ranging from 0.5 m to 10.0 m were tested. δd and δh were assumed to follow a zero-mean Gaussian distribution with standard deviations of 1 cm. δθ was assumed to follow a zero-mean Gaussian with 1.0 degrees of standard deviations. $\sigma_{\nabla \Delta \hat{N}}$ was set to 0.1 cycles.

Table 1 lists the computed $N_{avg}$ and confirms that it increased as the baseline length increased. For the relatively long baseline lengths of 5.0 and 10.0 m, there were large differences between the maximum and the minimum values of $N_{avg}$. For example, when the baseline length was 10.0 m, the maximum value was 121, and the minimum was 4. Among the thirteen satellites, the $N_{avg}$ values of seven satellites were less than 10 epochs.

**Figure 5:**
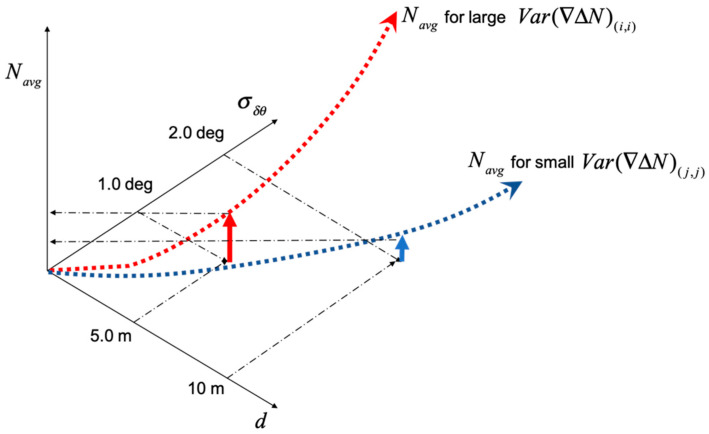
The figure describes the impact of *d* and $\sigma_{\delta \theta }^{}$ on $N_{avg}$ from Table 1 and Table 2. In general, $N_{avg}$ increases as *d* and $\sigma_{\delta \theta }^{}$ increase. However, the higher *d* and $\sigma_{\delta \theta }^{}$ do not always gurantee the larger $N_{avg}$ as shown above because $Var(\nabla \Delta \bar{N})$ also takes into account the directions of the double difference line-of-sight vector and $b_{0}$.

Table 2 lists the $N_{avg}$ values when the heading measurement accuracy degrades to 2.0 deg. The impact of the degraded heading measurement accuracy was minimal when the baseline length as less than 2.0 m. However, the impact became significant when the baseline lengths were larger than 5.0 m. Figure 5 illustrates the impact of *d* and $\sigma_{\delta \theta }^{}$ on the $N_{avg}$ from Table 1 and Table 2. As with Table 1, there was a large difference between the minimum and maximum values of $N_{avg}$ in the cases of 5.0 and 10.0 m baseline lengths. In this type of situation, rather than waiting for the maximum $N_{avg}$ epochs to resolve all of the integer ambiguities, a user can instead fix integer ambiguities of the subset of the satellites requiring a relatively smaller $N_{avg}$. The partially fixed integer ambiguities can provide an intermediate relative position solution that will be used to fix the integer ambiguities of the remaining satellites. This approach is referred to as partial integer ambiguity fix mode in this paper and is further described in the next subsection.

### 4.1. Partial Integer Ambiguity Fix Mode for Faster Integer Ambiguity Resolution

In the partial integer ambiguity fix mode, the required averaging epochs are recomputed by using the intermediate relative position obtained from the subset of satellites with lower $N_{avg}$. Assuming that the integer ambiguities of the subset of the satellites are correct and the position estimate with the subset of the satellites is ${\hat{x}}_{MF,PAR}$, the uncertainty of the position estimate, $P_{{\hat{x}}_{MF,PAR}}$, can be expressed as follows:

(16) $$P_{{\hat{x}}_{MF,PAR}}={\left(G_{PAR}^{T}W_{PAR}^{-1}G_{PAR}\right)}^{-1}.$$

**Figure 6:**
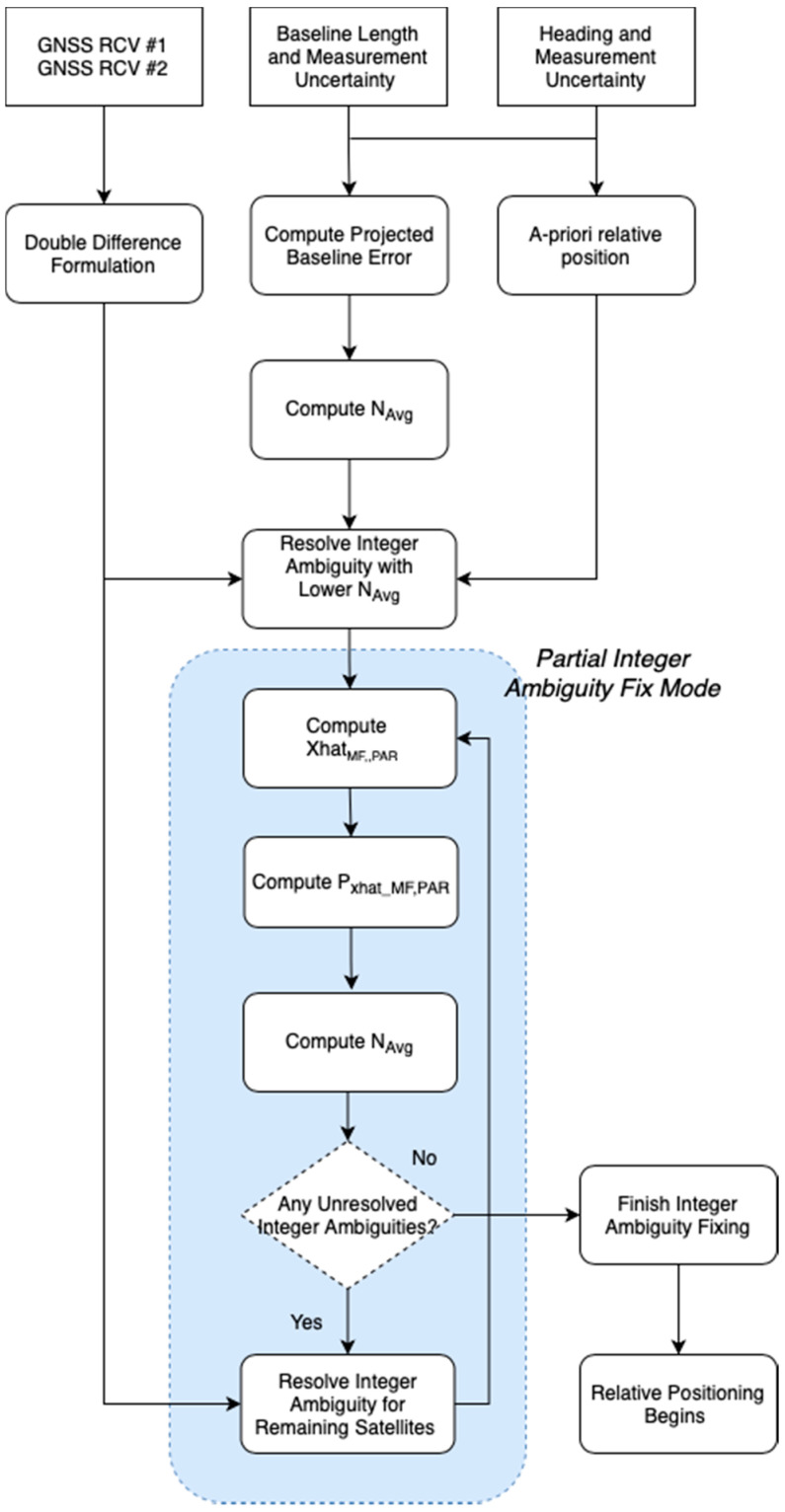
The overall integer ambiguity fix strategy with the partial integer ambiguity fix mode.

$G_{PAR}$ is the geometry matrix used to estimate ${\hat{x}}_{MF,PAR}$, and $W_{PAR}$ is the corresponding weighting matrix. Now, the integer ambiguities of the remaining satellites can be found using the variance of the double difference carrier phase in Equation (17) and averaging in Equation (18) as follows:

(17) $$Var(\nabla \Delta {\bar{N}}_{PAR})=\frac{G_{REM}\cdot P_{{\hat{x}}_{MF,PAR}}\cdot G_{REM}^{T}+Var(\nabla \Delta \bar{\varepsilon })}{\lambda^{2}},$$

where $G_{REM}$ is the geometry matrix for the remaining satellites. Then,

(18) $$\nabla \Delta {\hat{N}}_{avg,PAR}^{kl}={\left[\frac{1}{\lambda N_{avg,PAR}}\sum_{t=1}^{N_{avg,PAR}}\nabla \Delta \Phi_{MF}^{kl}(t)-(1_{M}^{k}(t)-1_{M}^{l}(t))\cdot {\hat{x}}_{MF,PAR}(t)\right]}_{roundoff},$$

where $N_{avg,PAR}$ is the number of averaging epochs computed using $Var(\nabla \Delta {\bar{N}}_{PAR})$ and Equation (15). As $Var(\nabla \Delta {\bar{N}}_{PAR})$ is small, $N_{avg,PAR}$ in the partial integer ambiguity fix mode becomes small as well. The immediate consequence is that the remaining integer ambiguities can now be fixed with much lower averaging epochs. Figure 6 shows the overall procedure of the proposed integer ambiguity fix strategies, including the partial integer ambiguity fix mode.

**Figure 7:**
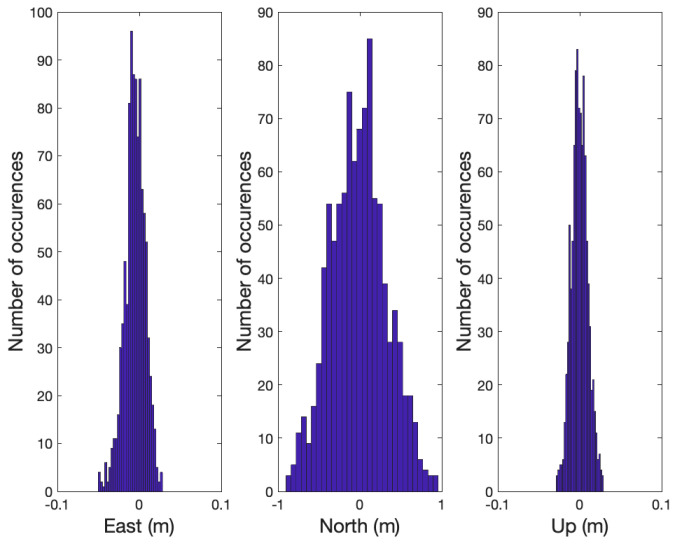
The figure shows the error distribution of the instantaneous a priori relative position, ${\hat{x}}_{MF,aprioi}$, in ENU coordinates determined by the launchpad and magnetometer in 1000 simulation cases.

To determine the effectiveness of the partial ambiguity fix, a total of 1000 cases were tested using the same simulation set-up for a 10 m baseline length with $\sigma_{\delta \theta }^{}$ = 2.0 deg in Table 2. Figure 7 shows the simulated instantaneous a priori relative position errors in which the follower positioning errors in the North were much larger than in East and Up due to inaccurate heading measurements and the relatively long baseline. In practice, it is recommended to apply a Kalman or an averaging filter to enhance the accuracy of the relative position before using it as ${\hat{x}}_{MF,aprioi}$. However, in order to access the performance of the proposed partial integer ambiguity with possible launchpad and magnetometer calibration errors, the instantaneous relative position shown in Figure 7 was used as ${\hat{x}}_{MF,aprioi}$.

**Figure 8:**
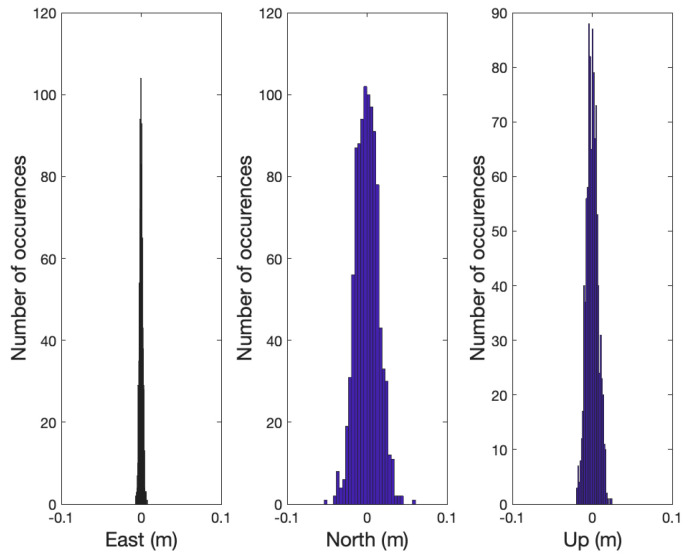
The figure shows the error distribution of the follower relative position, ${\hat{x}}_{MF,PAR}$, in ENU coordinates determined by the partially resolved integer ambiguities in 1000 simulation cases. The partial integer ambiguities were resolved at 16th epoch. ${\hat{x}}_{MF,PAR}$ was computed by averaging the position solutions with the carrier phase measurements during 16 epochs and the partially resolved integer ambiguities.

**Figure 9:**
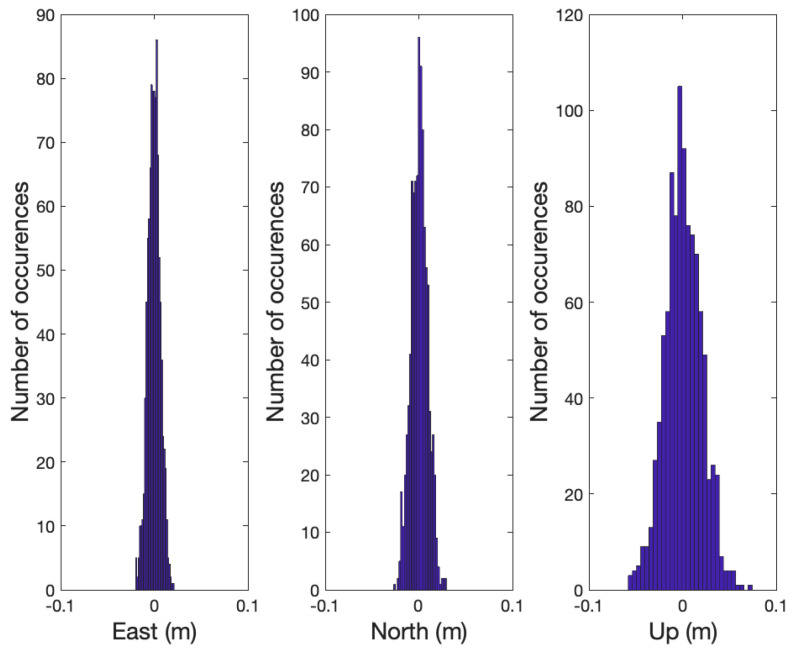
The figure shows the error distribution of the follower relative position in ENU coordinates after resolving the integer ambiguities of all satellites in 1000 simulation cases. All of the integer ambiguities were resolved at 16th epoch, and the position errors in the distribution were computed at the same epoch.

As the required averaging number of epochs, $N_{avg}$, is computed based on the double difference line-of-sight vectors and the covariance of ${\hat{x}}_{MF,aprioi}$, the $N_{avg}$ for all satellites remained constant as shown in Table 2 in the simulation. Therefore, the partial integer ambiguities of Sat 1, 3, 4, 7, 10, and 13 were fixed at the 16th epoch, and ${\hat{x}}_{MF,PAR}$ was computed with those satellites. Figure 8 shows the distribution of the averaged positioning errors in ${\hat{x}}_{MF,PAR}$. ${\hat{x}}_{MF,PAR}$ is averaged to reduce the multipath and noise effects assuming that the two GNSS receivers did not move. With ${\hat{x}}_{MF,PAR}$ and $P_{{\hat{x}}_{MF,PAR}}$, $N_{avg,PAR}$ for the remaining satellites was less than $N_{avg}$; therefore, the integer ambiguities of the remaining satellites could be immediately resolved. As a result, the required averaging epochs for all satellites was effectively $N_{avg}$, and no wrong integer fixes occurred in all 1000 cases. Figure 9 shows the distribution of the positioning errors using all satellites.

### 4.2. Positioning with Large Measurement Biases in A Priori Relative Position

The integer ambiguity fix methods proposed in the prior subsections work best if the a priori relative position is accurately measured. As discussed before, an accurate a priori relative position is resulted in the proposed launchpad set-up particularly when the heading measurement errors can be characterized as zero mean with small standard deviations. However, when the heading measurement errors have a bias, ${\hat{x}}_{MF,aprioi}$ and the corresponding integer ambiguity fix may have biases as well even with averaging.

Although a user may not be aware if the azimuth measurements have a bias or not, the integer ambiguity estimated through Equation (4) can be used to infer the possible presence of a large heading measurement bias from observing the fractional values of ${\nabla \Delta \hat{N}}_{avg}$. For example, all fractional values of ${\nabla \Delta \hat{N}}_{avg}$ will be around integer values when an accurate ${\hat{x}}_{MF,aprioi}$ is provided. However, as the biases in ${\hat{x}}_{MF,aprioi}$ become larger, the fractional values of some ${\nabla \Delta \hat{N}}_{avg}$ can drift with respect to time and could pass ±0.5 cycles, such that the integer ambiguity estimate changes.

To minimize the effect of large heading measurement biases, a weighting matrix can be used by applying the following rules. For example, if a satellite has ${\nabla \Delta N}_{avg}$ between ±0.4 and ±0.6 cycles, its ranging accuracy is designated as $100\lambda \sigma_{\nabla \Delta \varepsilon }$, where *λ* is the GNSS carrier phase wavelength, and $\sigma_{\nabla \Delta \varepsilon }$ is the standard deviation of the carrier phase multipath and noise in meters. The reason is that the heading measurement bias is assumed to cause up to one cycle deviation from the correct integer ambiguity. In addition, a float value of ${\nabla \Delta N}_{avg}$ is used for the position computation rather than being rounded off to an integer to avoid a roundoff error. The ranging accuracy of the remaining satellites is designated as $\sigma_{\nabla \Delta \varepsilon }$, and ${\nabla \Delta N}_{avg}$ is rounded to an integer. Since the heading measurement bias may cause a large bias in the integer ambiguity fix in a relatively long baseline length, a short baseline is recommended in this case.

## 5. Experimental Test Results

**Figure 10:**
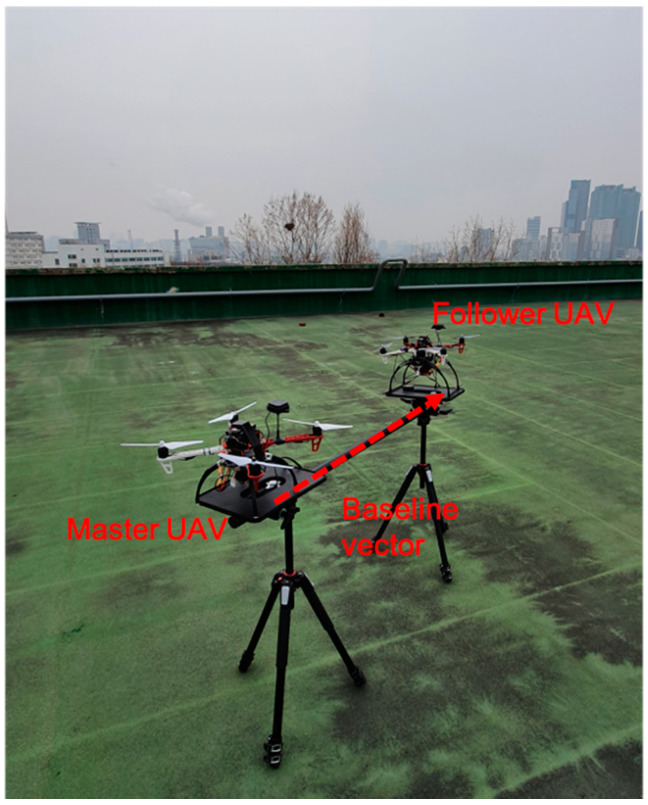
Proof-of-concept launchpad set-up for static tests at Hongik University, Seoul, Korea. Global Navigation Satellite System (GNSS) raw data were collected from two Ublox ZED-F9P receivers placed on the launchpad on 22 December 2020.

**Figure 11:**
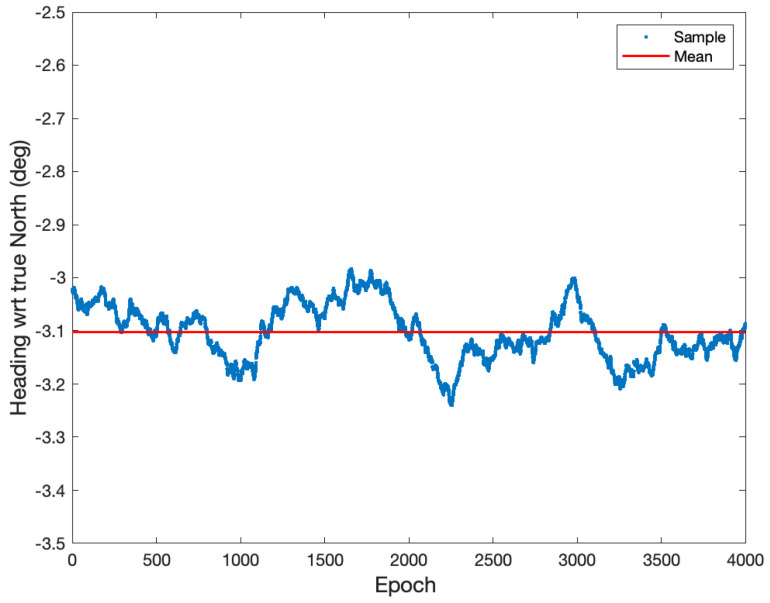
Heading measurements of the baseline shown in Figure 10. The measurements were obtained from a ST Micro LSM3030D magnetometer at a rate of 10 Hz. The mean value of the measurement data was −3.1 °, and the standard deviation was 0.05 °

The previous section details the processing algorithms, and this section presents the positioning performance of the proposed relative positioning method in a GNSS harsh environment. Figure 10 shows a launchpad testbed where two UAVs were placed with approximately 2 m separation during the test. Each UAV was equipped with Ublox ZED-F9P (Ublox, Thalwil, Switzerland)receivers and a Raspberry PI (Raspberry PI Foundation, Cambridge, England) to record the raw GNSS measurements. The heading direction from onboard the ST Micro LSM3030D (STMicroelectronics, Geneva, Switzerland) magnetometers was −3.7 degrees and was measured at 10 Hz. The heading measurement is shown in Figure 11.

**Figure 12:**
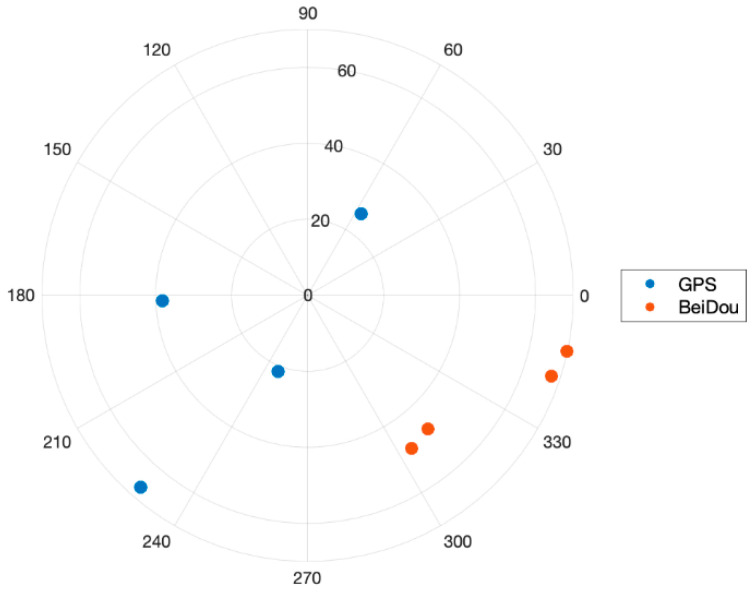
Sky plot of the GPS and BeiDou satellites taken at at Hongik University on 22 December 2020. There were a total of eight GPS and BeiDou satellites in view, which represents a GNSS harsh environment.

**Figure 13:**
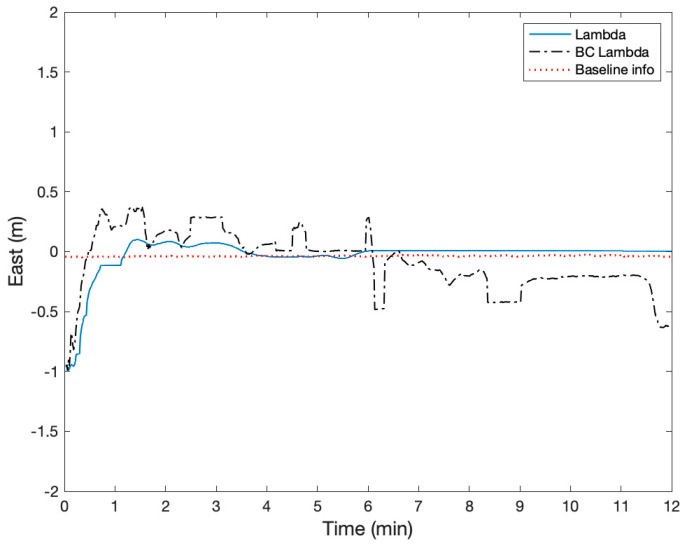
Comparison of the relative positioning error in the East using Least-squares Ambiguity Decorrelation Adjustment (LAMBDA), Baseline Constrained (BC) LAMBDA, and the proposed method using baseline information.

**Figure 14:**
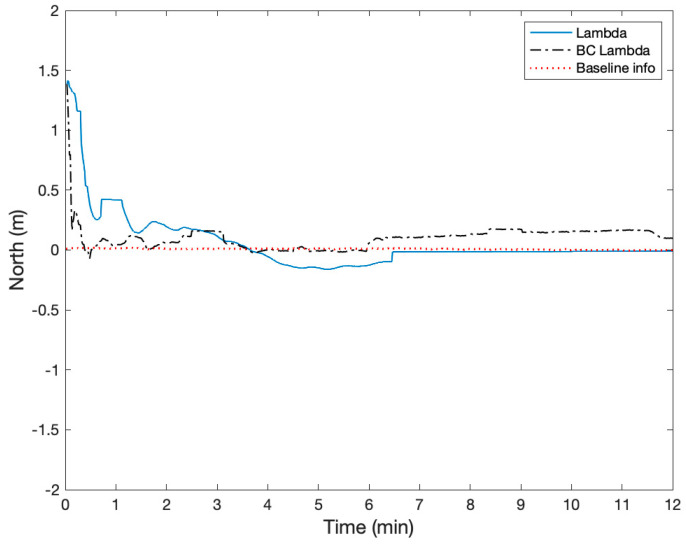
Comparison of the relative positioning error in the North using LAMBDA, Baseline Constrained (BC) LAMBDA, and the proposed method using baseline information.

**Figure 15:**
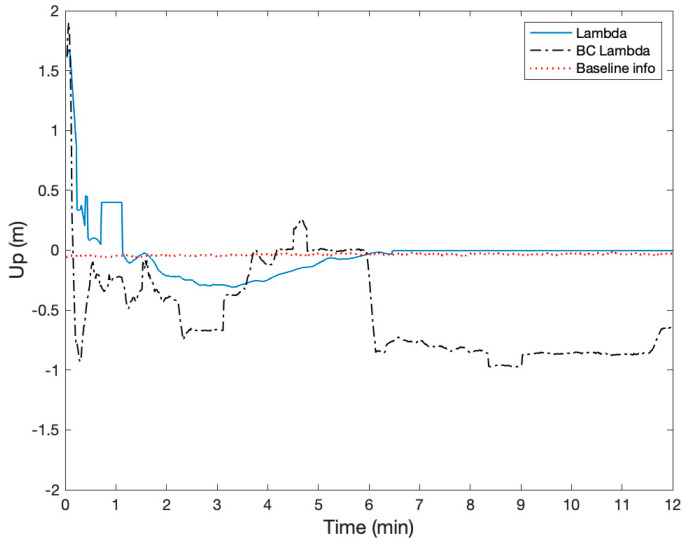
Comparison of the positioning error in the Up using LAMBDA, Baseline Constrained (BC) LAMBDA, and the proposed method using baseline information.

The height of two active antennas was calibrated to be on the same height using a laser level. During the static test, two Ublox ZED-F9P GNSS receivers were configured to output single-frequency GPS and BeiDou measurements at a rate of 1 Hz. The surveyed baseline relative position using a high-grade dual-frequency RTK system was −0.24, 2.02, and 0.02 m in East, North, and Up. Therefore, the true heading was −6.8 degrees with respect to North, and the heading error from the onboard magnetometer was about −3.1 degrees. The height difference of the two receivers was 1.6 cm. Figure 12 shows the GPS and BeiDou satellite locations, and there were four GPS and four BeiDou satellites. Figure 13, Figure 14 and Figure 15 compare the positioning errors of the conventional LAMBDA, baseline constrained LAMBDA, and the proposed method with the a priori baseline information.

The position solutions of the LAMBDA and the baseline constrained LAMBDA were obtained using RTKLIB [34]. In the proposed method, the maximum number of the averaging time was nine seconds after the partial ambiguity fix mode. The LAMBDA converged to a fixed solution after six minutes, and the baseline constrained Lambda never converged with relatively large position errors. The Root Mean Squares (RMS) of the positioning errors of the proposed method with the a priori baseline information were 3.7, 0.99, and 3.6 cm, and the position error characteristic was mostly a bias. There were no integer ambiguity candidates near ±0.5 cycles.

For the dynamic test, the follower receiver was installed on the roof of a car, and the master receiver was fixed on a tripod. The initial baseline vectors from the master receiver to the follower receiver were −5.56, −6.23, and −2.76 m in the East, North, and Up directions. Like the static test, single-frequency GPS and BeiDou measurements were used. In this test, a total of twenty one satellites were in view and some of the satellites were intentionally removed to compare the positioning performance based on LAMBDA with all satellites in view, LAMBDA with partial satellites in view, and the proposed method with partial satellites in view. The partial satellites in view consisted of a total of eight satellites, including four GPS and four BeiDou satellites. The heading measurement error was 1.2 degrees. Again, RTKLIB was used to obtain positioning solutions using LAMBDA methods.

**Figure 16:**
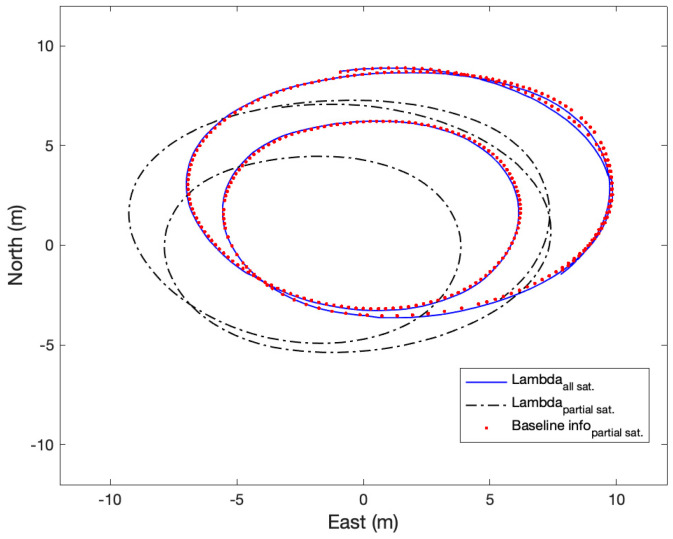
Comparison of the trajectory of a follower receiver using LAMBDA with all satellites in view, LAMBDA with partial satellites in view, and the proposed method with partial satellites in view. The LAMBDA solutions were obtained using RTKLIB. The test data were taken at the fourth industry revolution test site of Hongik University, Korea, on 12 September 2019.

With the proposed method, all of the integer ambiguities were resolved after 16 s in the test. Figure 16 shows the trajectories of the rover receiver from using LAMBDA with all satellites in view, LAMBDA with a partial satellite view, and the proposed method with a partial satellite view. While the trajectory of LAMBDA with all satellites in view and the proposed method with a partial satellite in view well matched, LAMBDA with the partial satellite view never reached a fixed solution and deviated from the other two solutions. Compared with LAMBDA with all satellites in view, the positioning errors of the proposed method with the partial satellites in view were 4.07, 11.03, and 13.12 cm, respectively. The slightly larger error of the dynamic test compared with the static test appeared to be caused by a small time delay in RTKLIB solutions.

## 6. Discussion

Through the simulations and short baseline field tests, the precise positioning capability of the proposed method was confirmed in the GNSS harsh environment with a small number of satellites in view. In the statics test, the maximum number of satellites used in the field tests was eight, which resulted in six single-frequency double difference measurements. In this environment, the proposed method significantly outperformed the position solutions of the LAMBDA and baseline constrained LAMBDA methods. The reason why the proposed method can provide near centimeter-level positioning capability with this low number of satellites in view is that most integer ambiguity in the double difference carrier phase measurements can still be accurately resolved through the aid of an a priori relative position. The dynamic test results also showed that the positioning performance of the proposed method with a small number of satellites in view is comparable with the LAMBDA using all satellites. Of course, the positioning capability of the proposed method will degrade as the accuracy of the a priori relative position degrades. To achieve the best performance, a fixed geometry, like the launchpad shown in Figure 1, with well calibrated magnetometers is recommended. Like other GNSS carrier-based positioning methods, cycle slips must be detected and remedied if possible.

While the proposed method performed well, an a priori relative position can be used to enhance the performance of the LAMBDA because the a priori relative position could be used instead of a float solution and would help LAMBDA to search for a smaller and proper set of integer candidates. This approach may resolve accurate integer ambiguities in a GNSS harsh environment even when a magnetometer has a large bias, which is our future research activity. Instead of using the proposed launchpad geometry, a priori relative position can be also obtained from using a precise distance measurement sensor like Ultra Wideband, which would lead to another form of a sensor fusion method.

## 7. Conclusions

The paper presented a novel GNSS carrier phase-based precise relative positioning method that used an a priori relative position to resolve the integer ambiguity. The a priori relative position allows observing the integer ambiguity in the double difference carrier phase measurements. Because the observed integer ambiguity also included noise and errors introduced by the imperfect a priori relative position, the proposed method used the iterative rounding scheme that sequentially resolved integer ambiguities based on the required averaging epochs. The formulations used to determine the required number of averaging epochs were also discussed in the paper. The static and dynamic field tests showed that the proposed method could provide a near centimeter-level positioning accuracy with a small number of satellites in view. In the same test environment, the LAMBDA and baseline constrained LAMBDA methods significantly underperformed in the aspects of a positioning accuracy and a time to first fix the integer ambiguity.

The contribution of this paper is how the GNSS carrier phase-based precise positioning can be accomplished in a GNSS harsh environment having a small number of satellites in view. Another benefit of the proposed method is that it can be applied for the low-cost single or dual frequency GNSS receivers. Although the set-up of the a priori relative position between two GNSS receivers may require a cumbersome procedure in general, a tool like the suggested launchpad would be helpful. The limitation of the proposed method is that the two GNSS receivers should maintain the a priori relative position until all of the integer ambiguities are resolved. Furthermore, another limitation might be the preferred short baseline of the a priori relative position within several meters. Therefore, the targeted application of the proposed method could be the formation flight of UAVs using a launchpad and an automatic take-off and landing of UAVs on cars or ships in a GNSS harsh environment.

## Figures and Tables

**Table 1:** The number of required averaging epochs to achieve $\sigma_{\nabla \Delta \hat{N}}$ of 0.1 cycles with five cases of baseline lengths. δθ was assumed to follow a zero-mean Gaussian distribution with 1.0 degrees of standard deviations.

Baseline Length	*d* = 0.5 m	*d* = 1.0 m	*d* = 2.0 m	*d* = 5.0 m	*d* = 10.0 m
Sat1	1	1	1	4	9
Sat2	1	4	4	25	64
Sat3	1	1	1	4	4
Sat4	1	1	1	4	4
Sat5	4	4	4	4	9
Sat6	1	4	4	16	64
Sat7	1	1	4	4	4
Sat8	1	4	4	9	36
Sat9	4	4	9	36	121
Sat10	1	1	1	4	4
Sat11	1	1	4	4	9
Sat12	4	4	4	16	49
Sat13	1	1	1	4	4

**Table 2:** The number of required averaging epochs to achieve $\sigma_{\nabla \Delta \hat{N}}$ of 0.1 cycles with five cases of baseline lengths. The simulation set up is identical to the one used in Table 1 except for $\sigma_{\delta \theta }^{}$= 2.0°.

Baseline Length	*d* = 0.5 m	*d* = 1.0 m	*d* = 2.0 m	*d* = 5.0 m	*d* = 10.0 m
Sat1	1	1	4	9	16
Sat2	4	4	16	64	225
Sat3	1	1	4	4	9
Sat4	1	1	4	4	9
Sat5	4	4	4	9	36
Sat6	4	4	16	64	196
Sat7	1	4	4	4	16
Sat8	4	4	9	36	121
Sat9	4	9	25	121	484
Sat10	1	1	4	4	4
Sat11	1	1	4	9	25
Sat12	4	4	16	49	169
Sat13	1	1	4	4	4

## References

[1] Montenbruck O., Ebinuma T., Lightsey E.G., Leung S. (2002). A Real-Time Kinematic GPS Sensor for Spacecraft Relative Navigation. Aerosp. Sci. Technol..

[2] Peyret F., Betaille D., Hintzy G. (2000). High-Precision Application of GPS in the Field of Real-Time Equipment Positioning. Autom. Constr..

[3] Olsen E.A., Park C.-W., How J.P. (1999). 3D Formation Flight Using Differential Carrier-Phase GPS Sensors. Navigation.

[4] De Jonge P., Tiberius C. (1996). Integer ambiguity estimation with the LAMBDA method. Proceedings of the GPS Trends in Precise Terrestrial, Airborne, and Spaceborne Applications.

[5] Li B., Verhagen S., Teunissen P.J.G. (2013). GNSS Integer Ambiguity Estimation and Evaluation: LAMBDA and Ps-LAMBDA. Proceedings of the China Satellite Navigation Conference (CSNC).

[6] Teunissen P.J.G. (2006). The LAMBDA Method for the GNSS Compass. Artif. Satell..

[7] Bakula M. (2020). Precise Method of Ambiguity Initialization for Short Baselines with L1-L5 or E5-E5a GPS/GALILEO Data. Sensors.

[8] Bakula M. (2020). Instantaneous Ambiguity Reinitialization and Fast Ambiguity Initialization for L1-L2 GPS Measurements. Sensors.

[9] Liu Z. (2011). A New Automated Cycle Slip Detection and Repair Method for a Single Dual-Frequency GPS Receiver. J. Geod..

[10] Kim D., Song J., Yu S., Kee C., Heo M. (2018). A New Algorithm for High-Integrity Detection and Compensation of Dual-Frequency Cycle Slip under Severe Ionospheric Storm Conditions. Sensors.

[11] Dai Z., Knedlik S., Loffeld O. (2009). Instantaneous Triple-Frequency GPS Cycle-Slip Detection and Repair. Int. J. Navig. Obs..

[12] Teunissen P.J.G., Kleusberg A. (1998). GPS observation equations and positioning concepts. GPS for Geodesy.

[13] Verhagen S. (2004). Integer Ambiguity Validation: An Open Problem?. GPS Solut..

[14] Teunissen P.J.G., Verhagen S. (2009). The GNSS Ambiguity Ratio-Test Revisited: A Better Way of Using It. Surv. Rev..

[15] Verhagen S., Teunissen P.J.G., Odijk D. The Future of Single-Frequency Integer Ambiguity Resolution. Proceedings of the VII Hotine-Marussi Symposium on Mathematical Geodesy.

[16] Odolinski R., Teunissen P.J.G. (2017). Low-Cost, High-Precision, Single-Frequency GPS–BDS RTK Positioning. GPS Solut..

[17] Odolinski R., Teunissen P.J.G. (2016). Single-Frequency, Dual-GNSS versus Dual-Frequency, Single-GNSS: A Low-Cost and High-Grade Receivers GPS-BDS RTK Analysis. J. Geod..

[18] Jackson J., Saborio R., Ghazanfar S.A., Gebre-Egziabher D., Davis B. (2018). Evaluation of Low-Cost, Centimeter-Level Accuracy OEM GNSS Receivers.

[19] Lee J.Y., Kim H.S., Choi K.H., Lim J., Chun S., Lee H.K. (2016). Adaptive GPS/INS Integration for Relative Navigation. GPS Solut..

[20] Liu T., Li B. Single-Frequency BDS/GPS RTK with Low-Cost u-Blox Receivers. Proceedings of the 2017 Forum on Cooperative Positioning and Service (CPGPS).

[21] Park C., Teunissen P.J.G. (2009). Integer Least Squares with Quadratic Equality Constraints and Its Application to GNSS Attitude Determination Systems. Int. J. Control Autom. Syst..

[22] Giorgi G., Teunissen P.J.G., Buist P.J. A Search and Shrink Approach for the Baseline Constrained LAMBDA Method: Experimental Results. Proceedings of the Proceedings International Symposium GPS/GNSS.

[23] Wang B., Miao L., Wang S., Shen J. (2009). A Constrained LAMBDA Method for GPS Attitude Determination. GPS Solut..

[24] Liu X., Chen G., Zhang Q., Zhang S. (2017). Improved Single-Epoch Single-Frequency Par Lambda Algorithm with Baseline Constraints for the BeiDou Navigation Satellite System. IET Radar Sonar Navig..

[25] Zhang Q., Ma C., Meng X., Xie Y., Psimoulis P., Wu L., Yue Q., Dai X. (2019). Galileo Augmenting GPS Single-Frequency Single-Epoch Precise Positioning with Baseline Constrain for Bridge Dynamic Monitoring. Remote Sens..

[26] Wu S., Zhao X., Zhang L., Pang C., Wu M. (2019). Improving Reliability and Efficiency of RTK Ambiguity Resolution with Reference Antenna Array: BDS+ GPS Analysis and Test. J. Geod..

[27] Teunissen P.J. (1998). Success Probability of Integer GPS Ambiguity Rounding and Bootstrapping. J. Geod..

[28] Teunissen P.J.G. Least-Squares Estimation of the Integer GPS Ambiguities. Proceedings of the Invited Lecture, Section IV Theory and Methodology, IAG General Meeting.

[29] Misra P., Enge P. (2006). Global Position Systems: Signals, Measurements and Performance.

[30] Kaplan E., Hegarty C. (2005). Understanding GPS: Principles and Applications.

[31] Caruso M.J. Applications of Magnetic Sensors for Low Cost Compass Systems. Proceedings of the IEEE 2000. Position location and navigation symposium (Cat. No. 00CH37062).

[32] Livada B., Vujić S., Radić D., Unkašević T., Banjac Z. (2019). Digital Magnetic Compass Integration with Stationary, Land-Based Electro-Optical Multi-Sensor Surveillance System. Sensors.

[33] GPSoft, Satellite Navigation TOOLBOX 3.0 User’s Guide, 2003. https://gpsoftnav.com/products/satellite-navigation-satnav-toolbox-3-0/.

[34] Takasu T., Yasuda A. (2009). Development of the Low-Cost RTK-GPS Receiver with an Open Source Program Package RTKLIB. Proceedings of the International symposium on GPS/GNSS.

